# Where do we aspire to publish? A position paper on scientific communication in biochemistry and molecular biology

**DOI:** 10.1590/1414-431X20198935

**Published:** 2019-08-29

**Authors:** M.S. Baptista, M.J.M. Alves, G.M. Arantes, H.A. Armelin, O. Augusto, R.L. Baldini, D.S. Basseres, E.J.H. Bechara, A. Bruni-Cardoso, H. Chaimovich, P. Colepicolo, W. Colli, I.M. Cuccovia, A.M. Da-Silva, P. Di Mascio, S.C. Farah, C. Ferreira, F.L. Forti, R.J. Giordano, S.L. Gomes, F.J. Gueiros, N.C. Hoch, C.T. Hotta, L. Labriola, C. Lameu, M.T. Machini, B. Malnic, S.R. Marana, M.H.G. Medeiros, F.C. Meotti, S. Miyamoto, C.C. Oliveira, N.C. Souza-Pinto, E.M. Reis, G.E. Ronsein, R.K. Salinas, D. Schechtman, S. Schreier, J.C. Setubal, M.C. Sogayar, G.M. Souza, W.R. Terra, D.R. Truzzi, H. Ulrich, S. Verjovski-Almeida, F.V. Winck, B. Zingales, A.J. Kowaltowski

**Affiliations:** Departamento de Bioquímica, Instituto de Química, Universidade de São Paulo, São Paulo, SP, Brasil

**Keywords:** Scientific journals, Scientific editing, Pre-prints, Open access, Peer review

## Abstract

The scientific publication landscape is changing quickly, with an enormous increase in options and models. Articles can be published in a complex variety of journals that differ in their presentation format (online-only or in-print), editorial organizations that maintain them (commercial and/or society-based), editorial handling (academic or professional editors), editorial board composition (academic or professional), payment options to cover editorial costs (open access or pay-to-read), indexation, visibility, branding, and other aspects. Additionally, online submissions of non-revised versions of manuscripts prior to seeking publication in a peer-reviewed journal (a practice known as pre-printing) are a growing trend in biological sciences. In this changing landscape, researchers in biochemistry and molecular biology must re-think their priorities in terms of scientific output dissemination. The evaluation processes and institutional funding for scientific publications should also be revised accordingly. This article presents the results of discussions within the Department of Biochemistry, University of São Paulo, on this subject.

## Introduction

**Figure 1.**
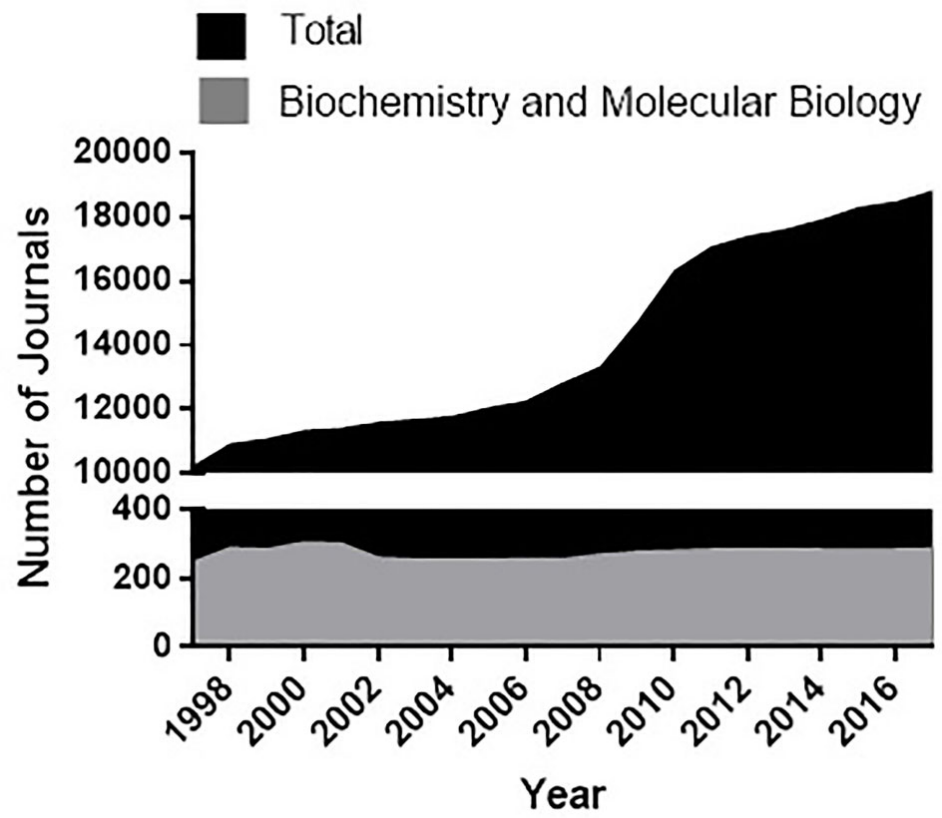
The number of academic journals has increased significantly over the last twenty years. Data were collected from the InCites Journal Citation Reports database from Clarivate Analytics on April 9, 2019. Numbers were unavailable for 2018.

Publishing in peer reviewed, internationally indexed, and widely read academic journals of good standing is considered the ideal mechanism to communicate scientific findings in our field. Although the number of journals related to biochemistry and molecular biology has remained relatively stable over the last decades, the total number of indexed academic journals has grown substantially (Figure 1). Indeed, biochemists and molecular biologists have increasingly sought to publish in general journals, as well as those of related areas that use biochemistry and molecular biology techniques. In view of this changing and growing academic editorial landscape, the Department of Biochemistry of the Institute of Chemistry, University of São Paulo, collectively discussed which journals are best suited to communicate our research.

Our focus within academic publishing is to produce and disseminate the best research possible. Good quality scientific publications involve specialized peer review in order to assure quality before showcasing the outcome widely. Scientific impact is often used for research evaluation processes, and impact assessment criteria should reflect quality publications. We thus believe that quality assessment must evolve to reflect the current perception of the best publication venues, and not primarily influence journal choice by authors. Furthermore, we believe guidelines can aid the choice of publication venues, but authors should always be free to make the final decision, as they are the most specialized persons in their particular field.

In Brazil, authors’ scientific output (including publications) can be found within the Lattes CV national research database (http://lattes.cnpq.br/), maintained by the Conselho Nacional de Desenvolvimento Científico e Tecnológico (CNPq), a federal research funding agency linked to the Ministry of Science, Technology, Innovation, and Communication (MCTIC). Online Lattes CVs list each researcher's publications, with DOIs, citation metrics for the journal (impact factor), and individual papers (number of citations), among many other academic activities.

Data on scientific productivity is collectively assessed by the Coordenação de Aperfeiçoamento de Pessoal de Nível Superior (CAPES), a federal government agency linked to the Ministry of Education responsible for ensuring quality graduate programs in Brazil by analyzing their research output. Nationally-recognized scores are attributed to each program based on these assessments, and programs may not be recommended or recognized by CAPES if minimal quality is not achieved. Quantifying scientific output (an important, albeit not the only criterion) involves separating publications into quality strata (using a CAPES scale named “Qualis”), which reflects their visibility and quality. Criteria vary in different scientific areas, but in biochemistry and molecular biology, they are mostly determined by journal impact factor. Impact factors are also often used internationally as a proxy to evaluate research output (1).

## Impact Factors

Impact factors are numerical values attributed by Thomson Reuters to academic journals that reflect the average number of times an article published in that journal is cited by other academic articles in a given year. The simplicity of this metric is probably what makes it by far the most used measure of journal prestige, despite the existence of other journal ranking systems such as the Eigenfactor and SCImago Journal Rank (2).

Impact factors have many caveats when used as metrics to evaluate the quality of a published article. Questions have been raised regarding the methods used by Thomson Reuters (3,4), which are not fully transparent, especially regarding what is considered an article by different journals. Other issues also remain, such as the fact that impact factors reflect the average number of citations per article, but citations vary widely for individual articles within the same journal. Most papers are cited significantly less than this average, since impact factors are usually raised by a very small number of highly cited papers (2,3). This should be sufficient to question impact factors as a quality evaluation metric. Indeed, the median number of citations of a journal article would be more meaningful as a measure of scientific visibility.

Additionally, journals have used strategies designed to increase impact factors, including the publication of a large number of review articles, creating commentaries and other sections that are not included as publications in impact factor calculations (but contribute toward journal citations), and even delaying final online publication dates versus preliminary online publication (5,6). Impact factors are also promoted by journal branding, in which new journals are created containing the “brand name” of an established and reputable journal in their title, and are therefore bolstered by the visibility and reputation inherited from the parent journal (7).

Overall, while impact factors are commonly used in academic publication assessment, we do not believe they should be the central determining factor when choosing publication venues. Instead, the audience reached by a journal, its time-tested reputation, type of publication, institutions backing it, and its editorial boards and policies should be prioritized.

## Academic Publications and Visibility

Many types of academic publishing formats exist today. We believe publishing in journals that perform stringent peer review is still the best manner to ensure scientific output quality. Many journals have evolved from print-only to electronic-only publication format (therefore reducing printing costs). In the digital era, we consider both publication formats equivalently visible for primary scientific publications.

Importantly, journals chosen for the communication of novel findings should be searchable in common indexing databases such as MedLine, PubMed, SCOPUS, and Web of Science so as to disseminate the findings widely. In this sense, book chapters, which have delayed publication and are often not indexed, are in disuse in our area for publishing novel findings (but may be appropriate for reviews and general scientific dissemination).

## Academic Publications: Pre-prints and Subscription *vs* Open Access Journals

Once published, full-text articles can be accessed by readers through different mechanisms. Traditionally, article access was purchased by libraries and institutions subscribing to the journal. Electronic access allowed for the addition of individualized post-payment options, including pay-per-article.

In the 1990s, powered by the advent of the World Wide Web, a new form of scientific publication was discussed, in which the group that was responsible for producing the results also covered publication costs, thus allowing open reading by anyone (now known as open access) (8). In 1991, a web-based and fully open access venue for scientific articles was inaugurated at arXiv.org, a collection in which manuscripts are self-archived by authors, showcased, criticized, and updated in an open manner (9). Archiving pre-prints was widely adopted early on in physics, but was not used by most biology researchers until recently. This was both due to lack of knowledge and tradition and because many biology journals did not allow prior distribution of a manuscript in archival form. This scenario has changed substantially over the last years, in parallel with the development of a pre-print server for biology, biorxiv.org, operated by Cold Spring Harbor Laboratory.

Due to the immediate dissemination of scientific results, pre-printed articles can gain attention before their final publication and be openly read in pre-print form, independent of the payment method of the journal that they are later published in. This is advantageous in terms of scientific dissemination and adheres to recommendations from the funding agency Fundação de Amparo è Pesquisa do Estado de São Paulo (FAPESP, Portaria CTA No. 01/2019).

However, some disadvantages exist for pre-prints within the biomedical area. Particularly for studies that involve possible clinical impact, pre-printing without the stringent control of peer review may be unadvisable (10). Even for studies that do not have clinical implications, a final publication in a peer-reviewed journal with good visibility is still considered an essential and final step for disclosure of scientific results. We, therefore, encourage the use of archives but do not recommend pre-printing of articles alone without seeking final publications in peer-reviewed and indexed journals.

Recently, the type of payment option (pre-paid open access, or subscription and pay-per-article journals) of peer-reviewed journals has been intensely debated. In particular, a group of research funders in Europe have announced a radical push toward the open access option called “Plan S”, which aims to make all scientific publications open access by January 2021 (11,12). This plan has been supported by a number of public and private research organizations, but has also been strongly criticized, mostly because of its limiting nature and unrealistic immediacy for implementation in face of various unresolved problems with open access publishing (13–16).

While the open access publication format has the unquestionable advantage of making scientific findings fully accessible to readers anywhere and should be a long-term goal for scientific publishing, it has also created problems. Because fees are covered entirely by grants awarded to authors, journals do not depend on publications with sufficient quality to sell to interested third parties, but only of sufficient quality to convince authors of publication merit. In parallel, authors are under pressure to publish in order to advance their careers and increase their competitiveness in grant applications to fund future research. The combination of these realities has resulted in a rapid increase in predatory journals, periodicals with little or no quality standards in which any paid content is published (17–21).

**Figure 2.**
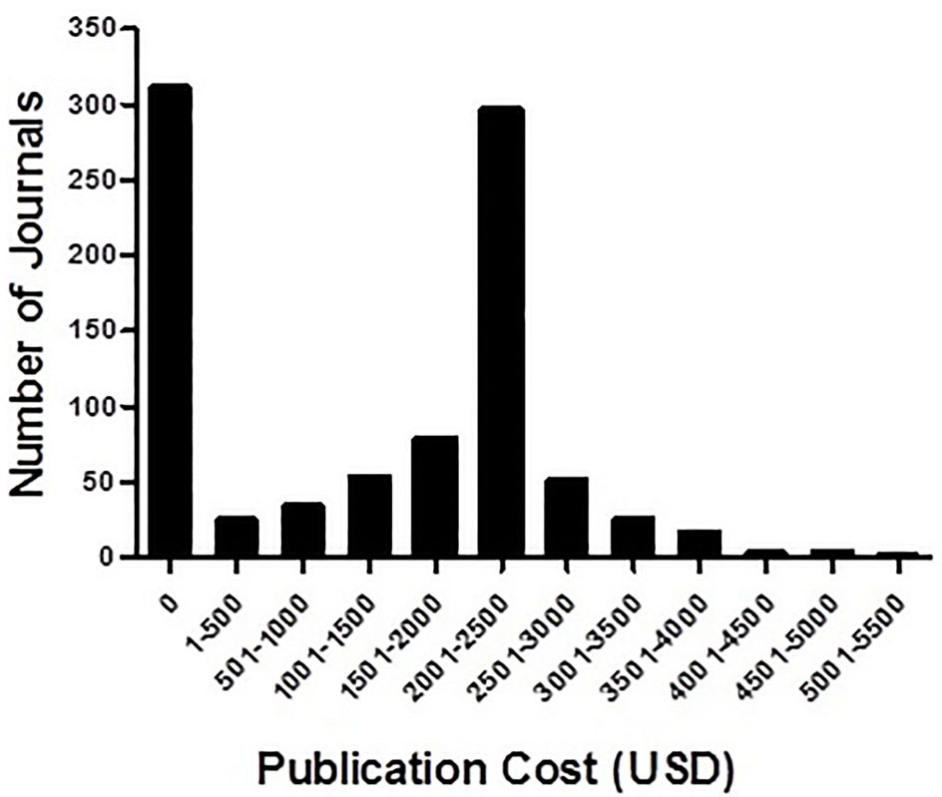
Price distribution for open access journals. Data were collected from online price lists for journals managed by Elsevier, Nature Publishing Group, Wiley, BioMed Central, and the Public Library of Science.

Another less discussed point about open access refers to the real cost of publishing and fair pricing. In a recent search for online prices in 894 open access journals from commercial editors (Figure 2), the median page charge was US$2145. Costs for authors varied from zero (many open access journals are maintained by means other than author charges, and are thus much less amenable to predatory practices (20)) to over US$5000 for a few periodicals. Since many for-profit open access-only publishers charge roughly the median price, this means that actual costs to maintain publications are around or below US$2000 per article. Therefore, prices that are much higher than this value seem highly questionable. Coincidentally or not, very high publication charges (US$5000 and over) are often practiced by brand-name “daughter journals” of high impact, well-recognized publications.

Scientific publishing does not obey market price rules, since authors cannot send papers to many journals to find the most cost-effective option. This results in profit margins well above average for these companies (22). Indeed, large institutions are increasingly refusing to pay the high prices some publishers charge for post-payment scientific articles (23,24). Within this scenario, editorial companies may use pre-payment in open access models to guarantee maintenance of high profits as a more secure payment form.

Another point pertaining to open access is the source of funding for payments. In Brazil, journal contents are widely accessible due to the CAPES Periodicals Portal, which subscribes to over 45,000 full-text international journals for research and academic institutes in the whole country. On the other hand, open access payment is the authors’ responsibility. While most research grants cover page charges, there is no supplementary financing for open access charges, and authors must therefore choose between paying for open access and consumables for their research (14 [Bibr B15]). In the long run, if open access is to be prioritized, current CAPES funding for subscriptions should somehow be diverted to cover open access publication costs.

Overall, we consider open access publication interesting and desirable in the long run, based on the principle that most academic research is publicly funded, and therefore the public should have access to the results. However, given the current lack of control over quality and pricing in this model, as well as the lack of a secure funding source that does not compromise ongoing research efforts, we do not believe open access publishing should be the primary decisive point when selecting where to publish. We remind authors and readers that full text versions of articles can always be exchanged among scientists through personal correspondence, independently of the journal payment model.

## Academic Publications: Editorial Organization

Perhaps more important than the payment form in publishing is re-thinking the destiny of profits and their potential application towards future scientific endeavors. There are three general kinds of organizations for editorial publishing: journals that are edited by scientific societies, journals that are maintained by private companies, and journals that are maintained by private companies in partnership with scientific societies. The models that involve scientific societies result in returns of at least part of the editorial revenue to the scientific community. This revenue is often used to fund fellowships, meetings, and other scientific activities. On the other hand, journals that are not backed by scientific societies are free to do as they wish with their profits. A strong link with scientific societies also guides the editorial decisions of a journal, steering it toward academically-relevant, rather than economically-relevant, decisions.

Additionally, scientists can participate in journals by acting as academic editors (who select peer-reviewers), within editorial boards (the researchers most often consulted as reviewers and who help determine the journal's focus points), and as advisory panels or steering committee members. Researchers unaffiliated with the journal are also recruited to review manuscripts, and typically do so voluntarily, despite the fact that many editorial companies are for-profit. Some journals increasingly use professional editors instead of active scientists to choose and organize peer review, aiming to streamline the process. More recently, a trend for using professional editors as editorial board members has also emerged. In particular, Nature titles (excluding the journal Nature, but including over 50 different journals published by the group) have replaced active scientists on their editorial boards by full-time PhD-level professional editors.

While professional editors may be highly prepared, and having some level of professional editorial skills can help accelerate the revision process, we view the link with active academic scientists as crucial to maintain not only quality peer review but also adequate journal management over time. Overall, the reputation of a journal as well as the quality of the researchers who act in positions within the journal should be prioritized when choosing venues for academic publishing. Journals published by or linked to strong scientific societies are of particular interest, since this guarantees quality maintenance over time and provides a mechanism to return profits from editorial actions back to scientific activities.

## Overview

Within the changing landscape of scientific publishing, the authors of this manuscript aspire to promote quality manuscript publications by prioritizing the following actions:

- Submit pre-prints whenever possible, avoiding their use only when a target journal does not permit it or it is unadvisable (such as for clinical studies), and then proceed with submission to a quality peer-reviewed journal.
- Value and provide high quality peer-review in scientific journals, both by delivering careful revisions, when requested, and seeking good revisions of submissions.
- Give preference to journals with a solid and time-tested reputation for quality, irrespective of current impact factor.
- Denounce and avoid unfair pricing for both subscription and open access journals.
- Value journals with good visibility, indexed widely, and that appeal to a general audience.
- Give preference to journals with strong links to academic societies and that include active and highly reputable investigators on their boards and advisory committees.

In providing these guidelines for our aspired publication venues with a strong focus on quality, we hope institutions (universities, research institutes, and funding agencies) will follow by adapting their assessment criteria accordingly. A concerted effort from both researchers and institutions as well as constant revisions of guidelines such as the ones presented herein will hopefully allow the scientific community to successfully navigate the fast changes that are occurring in mechanisms to showcase novel scientific discoveries.
